# Crystal Structures of 6-Phosphogluconate Dehydrogenase from *Corynebacterium glutamicum*

**DOI:** 10.4014/jmb.2305.05002

**Published:** 2023-06-27

**Authors:** Hyeonjeong Yu, Jiyeon Hong, Jihye Seok, Young-Bae Seu, Il-Kwon Kim, Kyung-Jin Kim

**Affiliations:** 1 School of Life Sciences, BK21 FOUR KNU Creative BioResearch Group, Kyungpook National University, Daegu 41566, Republic of Korea; 2 KNU Institute for Microorganisms, Kyungpook National University, Daegu 41566, Republic of Korea

**Keywords:** 6-Phosphogluconate dehydrogenase, *Corynebacterium glutamicum*, crystal structure, 6-phosphogluconate, nicotinamide adenine dinucleotide phosphate

## Abstract

*Corynebacterium glutamicum* (*C. glutamicum*) has been considered a very important and meaningful industrial microorganism for the production of amino acids worldwide. To produce amino acids, cells require nicotinamide adenine dinucleotide phosphate (NADPH), which is a biological reducing agent. The pentose phosphate pathway (PPP) can supply NADPH in cells via the 6-phosphogluconate dehydrogenase (6PGD) enzyme, which is an oxidoreductase that converts 6-phosphogluconate (6PG) to ribulose 5-phosphate (Ru5P), to produce NADPH. In this study, we identified the crystal structure of 6PGD_apo and 6PGD_NADP from *C. glutamicum* ATCC 13032 (*Cg*6PGD) and reported our biological research based on this structure. We identified the substrate binding site and co-factor binding site of *Cg*6PGD, which are crucial for understanding this enzyme. Based on the findings of our research, *Cg*6PGD is expected to be used as a NADPH resource in the food industry and as a drug target in the pharmaceutical industry.

## Introduction

*Corynebacterium glutamicum* (*C. glutamicum*), which is a Gram-positive aerobic bacterium that grows in environment with sugars and organic acids, was isolated in 1957 by Kinoshita and his coworkers [1]. *C. glutamicum* provides nucleotides, chemicals, materials, biofuels, and amino acids [2, 3]. Because of its properties, it has been widely used as an important industrial microorganism in the production of amino acids for a long time [4]. There are many *C. glutamicum* strains; among them, *C. glutamicum* ATCC 13032 has been investigated, to develop efficient production strains [5]. In the application of *C. glutamicum* as an essential industrial microorganism, most of the strains require nicotinamide adenine dinucleotide phosphate (NADPH). In other words, a large amount of NADPH cofactors are required as reducing power for amino acid synthesis [6]. Because the pentose phosphate pathway (PPP) can supply NADPH in cells, PPP in *C. glutamicum* is very important. Therefore, recently, intensive research has been carried out on the PPP in *C. glutamicum*, to enhance the production of L-lysine, L-glutamate-family amino acids (GFAAs), L-leucine, L-valine and L-isoleucine [7-13].

The PPP, which is the essential pathway in cellular metabolism, provides a precursor for nucleotide or amino acid synthesis and protects cells against oxidative stress. The PPP can be divided into two phases : the oxidative phase (OPPP) and the non-oxidative phase (NOPPP). During the oxidative phase, NADPH is produced from glucose using the reducing power of glucose-6-phosphate dehydrogenase (G6PDH) and 6-phosphogluconate dehydrogenase (6PGD). These two isoenzymes play an important role in this pathway in terms of the production of NADPH, which is an essential co-factor to the survival of cells and a biological reducing agent that is used in the synthesis of fatty acids and cholesterol [14]. Moreover, many studies have indicated that NADPH is a key factor in cellular antioxidation systems and oxidative stress [15]. However, cells contain more NAD than NADP. Therefore, a suitable intracellular NADPH level is important for maintaining the redox balance in cells [16]. To reduce this imbalance of energy-carrying molecules in cells, many researchers have attempted to replace NADP-dependent enzyme with NAD-dependent enzyme. For this reason, G6PDH and 6PGD, which can produce NADPH, are important. These enzymes react with NADP, rather than NAD, thus easily offering NADPH in cells.

**Fig. 1:**
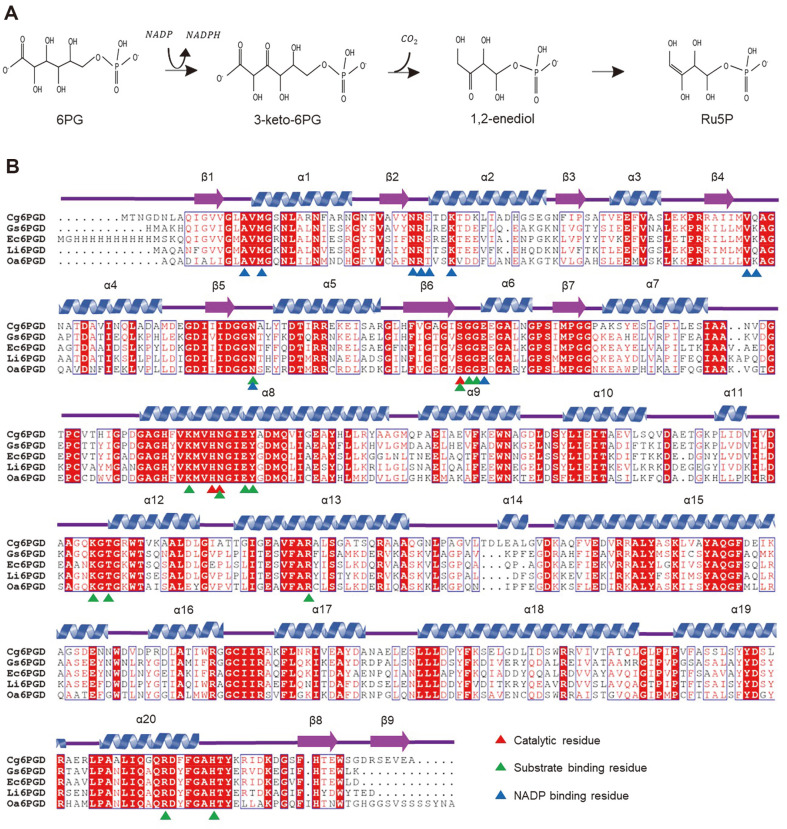
Reaction and sequence alignment of *Cg*6PGD. (**A**) Enzymatic reaction of 6-phospho gluconate dehydrogenase. NADPH and CO_2_ are produced from this reaction. (**B**) Amino acid sequence alignment of *Cg*6PGD with other 6PGD structures. The secondary structural elements are drawn based on the structure of *Cg*6PGD and labeled. Residues that were involved in catalytic activity, substate-binding, and NADP-binding are presented using differently colored triangles, respectively.

Furthermore, 6PGD, which catalyzes the conversion of 6-phosphogluconate (6PG) to ribulose 5-phosphate (Ru5P) using NADP in the third step of PPP, to produce NADPH (Fig. 1A) [17], is well known as a drug target in cancer and infection. Studies on the control of 6PGD revealed that this causes a decrease in lipogenesis and RNA biosysnthesis and an increase in ROS levels, consequently hindering the growth of cancer cells [18] . Moreover, 6PGD plays an important role in the metabolic system, but also acts as a potential drug target for African trypanosomes [19, 20]. The structures of the 6PGD-based amino acid sequences from *Geobacillus stearothermophilus*, *Escherichia coli* K-12, *Lactococcus lactis* and *Ovis aries* have been determined (Fig. 1B).

The present research focused on the 6PGD from *C. glutamicum* ATCC 13032 (*Cg*6PGD), which is composed of 484 amino acids. We determined the crystal structure of the *Cg*6PGD enzyme in the apo form (*Cg*6PGD_apo) and in the complex form with the NADP co-factor (*Cg*6PGD_NADP). We also report the properties of *Cg*6PGD based on structural analysis and biochemical studies.

## Materials and Methods

### Expression and Purification

The *Cg*6PGD (Cgl1452, NCg11396) gene was amplified from the genomic DNA of *C. glutamicum* ATCC 13032 by polymerase chain reaction (PCR) with Pfu polymerase (ELPISBIOTECH, Korea). The amplified PCR product was subcloned into the pET-30a expression vector, to incorporate a C-terminal (His)_6_ tag in the corresponding protein product. pET-30a:*Cg*6PGD was transformed into the *Escherichia coli* strain BL21(DE3)-T1^R^. The transformed cells were cultured in 2 L of Luria Bertani (LB) medium containing 50 mg l^-1^ kanamycin at 37°C. At an Optical Density at 600 nm of 0.6, the cells were induced with 0.5 mM isopropyl β-D-1-thiogalactopyranoside (IPTG). After incubation for 18-20 h at 18°C with shaking at 120 rpm, the cells were harvested by centrifugation at 4,000 rpm for 15 min at 20°C. Subsequnetly, the cell pellet was resuspended in buffer A (40 mM Tris-HCl, pH 8.0) and cell lysis was achieved via ultrasonication. The cell debris was removed by centrifugation at 13,000 rpm for 30 min and the supernatant was applied onto an Ni-NTA agarose column (Qiagen, Germany) that had been pre-equilibrated with buffer A. After washing with buffer A containing 27 mM Imidazole, the bound proteins were eluted with 300 mM Imidazole in buffer A. The final yield of *Cg*6PGD was 45.3 mg per liter of LB. After Ni-NTA purification, size-exclusion chromatography was performed using a HiPrep 26/60 Sephacryl S-300 HR column (320 ml; Cytiva, USA) that had been pre-equilibrated with buffer. All purification experiments were performed at 4°C. Finally protein purity was confirmed by sodium dodecyl sulfate-polyacrylamide gel electrophoresis (SDS-PAGE).

### Crystallization

After the purification of the *Cg*6PGD protein, crystallization was initially performed using the sitting-drop vapor diffusion method at 20°C. Crystallization was performed using the commercially available sparse-matrix screens, including Wizard Classic I and II, CRYO I and II (Rigaku Reagents, USA), Index, PEG/ION I and II (Hampton Research, USA), and Structure Screen I and II (Molecular Dimensions), via the hanging-drop vapor diffusion method at 20°C.

Each experiment consisted of mixing 1.0 ul of protein solution (41.25 mg ml^–1^ in 40 mM Tris-HCl, pH 8.0) with 1.0 ml of reservoir solution and equilibrating the drop against 50 ul of reservoir solution. The crystals of *Cg*6PGD were observed under various crystallization screening conditions. The best diffracting crystal of *Cg*6PGD_apo was formed in a reservoir solution consisting of 10% (w/v) polyethylene glycol 8000, 0.1 M Tris-HCl pH 7.0 and 0.2 M magnesium dichloride. The crystallization screening method used for *Cg*6PGD complexed with NADP (*Cg*6PGD_NADP) was the same as that employed for *Cg*6PGD, with the exception of the addition of 8 mM NADP to the protein solution. In turn, the best diffracting crystal of *Cg*6PGD_NADP appeared in a reservoir solution consisting of 15% (w/v) polyethylene glycol 3350 and 0.3 M magnesium formate dihydrate.

### Data Collection and Structure Determination

The best quality *Cg*6PGD crystals were transferred to a cryoprotectant solution containing 30% (v/v) glycerol. The crystals were harvested with a loop of 0.3 mm diameter and flash-frozen by immersion in liquid nitrogen at -173°C. The X-ray diffraction data were obtained at the 7A beamline of the Pohang Accelerator Laboratory (PAL, Republic of Korea) using a Quantum 270 CCD detector (ADSC, USA) [21]. The crystals of *Cg*6PGD_apo and *Cg*6PGD_NADP diffracted to the resolutions of 2.4 and 1.9 Å, respectively. All data were indexed, integrated, and scaled together using the HKL2000 software suite [22]. The *Cg*6PGD_apo belonged to the space group P 2_1_ 2_1_ 2_1_ with unit cell parameters of *a* = 63.9 Å, *b* = 120.3 Å, *c* = 152.6 Å, and α = β = γ = 90.0°. With two molecules of *Cg*6PGD per asymmetric unit, the Matthews coefficient was 2.83 Å^3^ Da^-1^ , corresponding to a solvent content of approximately 56.62%. The *Cg*6PGD_NADP belonged to the space group P 2_1_ 2_1_ 2_1_ with unit cell parameters of *a* = 64.0 Å, *b* = 119.47 Å, *c* = 153.45 Å, and α = β = γ = 90.0°. With two molecules of *Cg*6PGD per asymmetric unit, the Matthews coefficient was 2.83 Å^3^ Da^-1^ , corresponding to a solvent content of approximately 56.64% [23]. Both structures were determined using the CCP4 version of MOLREP through molecular replacement, with 6PGD from *Geobacillus stearothermophilus* (PDB code 2W8Z, 56.1% sequence identity) utilized as a search model [24, 25]. Model building was performed manually using the WinCoot program, and refinement was carried out using CCP4 refmac5 [26-28]. The refined models of *Cg*6PGD_apo and *Cg*6PGD_NADP were deposited in the Protein Data Bank with PDB codes of 8I4N and 8I4Q, respectively (Table 1).

### Analytical Size-Exclusion Chromatography

To determine the oligomeric status of *Cg*6PGD, we performed analytical size-exclusion chromatography using a Superdex 200 Increase 10/300 GL column (Cytiva). A protein sample of 1 ml at a concentration of 1 mg ml^–1^ was analyzed with equilibrium buffer (40 mM Tris-HCl, pH 8.0, and 150 mM NaCl). The molecular mass of the purified *Cg*6PGD sample was calculated using a calibration curve, which was constructed using ferritin (440 kDa), aldolase (158 kDa), ovalbumin (44 kDa), and ribonuclease (13.7 kDa) standard samples.

### Amino Acid Sequence Analysis of Substrate Binding Site

The amino acid sequence analysis was performed using a position-specific iterated basic local alignment search tool (PSI-BLAST). For this, 705 *Cg*6PGD homologs with an identity exceeding 98% and a query cover exceeding 65% were selected, excluding partial data. Multiple sequence alignment was performed using Clustal Omega software [29]. A conservation analysis was performed using WebLogo [30].

### Molecular Docking Simulation

A simulation of the molecular docking of 6PG to the *Cg*6PGD structure was carried out using AutoDock Vina software [31]. The ligand molecules of 6PG was prepared using the Protein Data Bank (PDB) website, pdbqt files were generated using AutoDock tools, and the process used for molecular docking simulation was carried out according to the AutoDock Vina manual. Flexible residues (Tyr195, Lys265, Arg292 and Arg454) were selected for the generation of pdbqt files for both rigid and flexible receptors. The residues mentioned above were selected based on the co-crystallization structure obtained from *Geobacillus stearothermophilus* (PDB code 2W8Z) [32].

The grid box size for *Cg*6PGD was x = 102, y = 90, z = 122; and the grid center was set at x = 9.076, y = -20.37, z = -27.629. Nine output poses were generated and their free energy of binding was calculated from their own scoring function. The final poses of 6PG docked into *Cg*6PGD were selected based on the substrate binding site of *Cg*6PGD using the PyMOL software. The affinity of the predicted docking result was -7.3 kcal/mol.

### Enzymatic Activity Assay

The Enzymatic activity of *Cg*6PGD was determined using ultraviolet-visible (UV-Vis) spectroscopy. The enzymatic activity was measured from the forward reaction rate of 6PG by monitoring the NADPH level at a wavelength of 340 nM (extinction coefficient of approximately 6,220 M^–1^ cm^–1^) [33]. The reaction mixture included 40 mM Tris-HCl buffer (pH 8.0), 2 mM NADP or 5 mM 6PG, and 0.2 uM *Cg*6PGD protein. All enzymatic assays were carried out at 20°C in a final volume of 500 ul in duplicate. The kinetic parameters were obtained by measuring the initial velocity with the concentration of 6PG varying from 0.1 to 5 mM while keeping that of NADP constant at 2 mM, to check the substrate affinity. In addition, the initial velocity was determined at a concentration of NADP varying from 0.01 to 2 mM while keeping that of 6PG constant at 5 mM, to check the affinity of the co-factor. The *V_max_* and *K_m_* values were determined using Michaelis-Menten equation, and the results were plotted as a graph using the OriginPro program (OriginLab Corporation, USA).

## Results and Discussion 

### Overall Structure of *Cg*6PGD

We determined that the crystal structures of *Cg*6PGD_apo and *Cg*6PGD_NADP to elucidate the molecular mechanism of *Cg*6PGD. The refined structures have a good agreement with the X-ray crystallographic statistics (Table 1). An NCBI BLAST analysis using the PDB revealed that many data entries had a high similarity to, and high query coverage with *Cg*6PGD. The overall structure of *Cg*6PGD was similar to that of 6PGD of *Geobacillus stearothermophilus* (*Gs*6PGD, Uniprot entry I3NI58, PDB 2W8Z) with an r.m.s.d. value of 0.842 Å and 56.1%sequence identity, *Escherichia coli* K-12 (*Ec*6PGD, Uniprot P00350, PDB 3FWN) with r.m.s.d. value of 1.257 Å and 54.43% sequence identity, *Lactococcus lactis* (*Li*6PGD, Uniprot P96789, PDB 2IYO) with r.m.s.d. value of 1.443 Å and 52.13% sequence identity, and *Ovis aries* (*Oa*6PGD, Uniprot P00349, PDB 1PGN) with r.m.s.d. value of 0.920 Å and 49.04% sequence identity. Moreover, they were similar in sequence and structure, and various important sequences were well conserved (Fig. 1B). However, remarkable structural differences are observed in the NADP binding site. Compared to the other 6PGD structures, the loop at α10-α11 region moved slightly away from the NADP binding site in *Cg*6PGD. We suspect that the movement makes the NADP binding pocket more accessible for NADP to bind more easily.

**Fig. 2:**
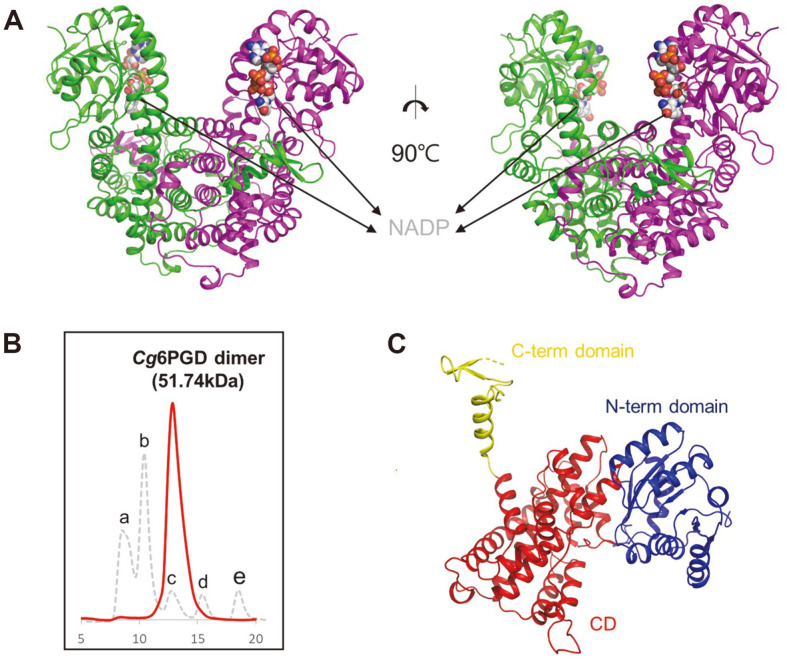
Overall structure of *Cg*6PGD. (**A**) Dimeric structure of *Cg*6PGD. The dimeric structure is shown as a cartoon model, and the two chains are distinguished with different colors : green and magenta, respectively. NADP is presented as a gray-colored sphere model. (**B**) Size-exclusion chromatography of *Cg*6PGD. a is a void peak, b is ferritin (440 kDa), c is aldolase (158 kDa), d is ovalbumin (44 kDa) and e is ribonuclease (13.7 kDa). *Cg*6PGD is eluted as a dimer. (**C**) Domain classification of *Cg*6PGD using a cartoon model of the monomer. The N-term domain is indicated in blue, the CD is indicated in red, and the Cterm domain is indicated in yellow.

*Cg*6PGD consists of twenty α-helices and twelve β-strands (Fig. 2A). The crystal forms of both structures included a dimer per asymmetric unit. To clarify the oligomeric status of these enzymes, we performed analytical size-exclusion chromatography. *Cg*6PGD eluted with a molecular weight of approximately 116.8 kDa, indicating that *Cg*6PGD exists as a dimer in solution (Fig. 2B).

The monomeric structure of *Cg*6PGD could be divided into three functional domains : the N-terminal domain (N-term domain, Met1-Pro179), the central domain (CD, Asp180-Arg441), and the C-terminal domain (C-term domain, Ala442-Ala484) (Fig. 2C). The N-term domain consists of seven β-strands surrounded by seven α-helices and was associated with NADP-binding. This domain adopted the Rossman fold with an additional α-β-α unit [34]. The CD consisted of α-helices exclusively and was associated with dimerization [17]. Finally, the C-term domain, also known as the tail domain, was located near central domain of neighboring chain.

### Substrate-Binding Site and Catalytic Residues

**Fig. 3:**
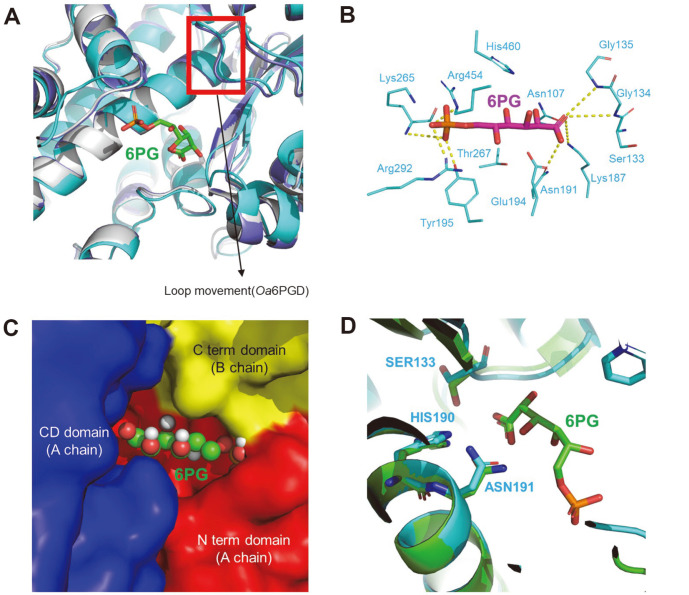
Substrate(6PG)-binding model of *Cg*6PGD. (**A**) Structure comparison between *Oa*6PGD_apo, *Oa*6PGD_6PG and *Cg*6PGD_apo. All structures are presented as cartoon diagrams. The structures are depicted in gray (*Oa*6PGD_apo), purple (*Oa*6PGD_6PG) and cyan (*Cg*6PGD). (**B**) Docking simulation of *Cg*6PGD with 6PG. Hydrogen bonds between the residues and 6PG are expressed, with the exception of water-meditated hydrogen bonds. 6PG is presented as a magenta-colored stick model, and the substrate-binding residue is presented as a cyan-colored stick model. All residues are labeled. (**C**) Domains associated with the binding of 6PG. (**D**) Catalytic residues of *Cg*6PGD and *Oa*6PGD. *Cg*6PGD is presented as a cyan-colored cartoon model, and *Oa*6PGD is presented as a green-colored cartoon model. The catalytic residues are presented using sticks and labeled.

The substrate-binding mode was predicted via the alignment of our structure with the *Oa*6PGD structure in the apo form (PDB code : 2PGD) and in complex with 6PG (PDB code : 1PGP). In *Oa*6PGD, the movement of residues triggered by substrate-binding is small in the 6PG complex [35]. Based on the structural similarity between *Oa*6PGD and *Cg*6PGD, we also expected that *Cg*6PGD would have small movements when combined with 6PG. This movements suspect to occur during dehydrogenation and conformational change caused by the binding of the 6PG would be small (Fig. 3A). The substrate-binding site of 6PGD is conserved and well-known. To predict the substrate-binding site of *Cg*6PGD, similar structures (*Gs*6PGD and *Ec*6PGD) were used to perform alignments, which revealed that the Asn107, Ser133, Gly134, Gly135, Lys187, Asn191, Glu194, Tyr195, Lys265, Thr267, Arg292, Arg454, and His460 residues were involved in the binding to substrate in *Cg*6PGD. Moreover, these residues were conserved in other 6PGD amino acid sequences (Fig. 1B). In turn, the Tyr195, Lys265, Arg292 and Arg454 residues stabilized the phosphate moiety of 6PG through hydrogen bonds, and the Asn191, Glu194 and Thr267 residues also formed a water-mediated hydrogen bond with the phosphate moiety of 6PG. In addition, Asn107, Ser133, Gly134, Gly135 and His460 established a water-mediated hydrogen bond with the remaining part of 6PG. Lys187 and Glu194 were critical residues for enzyme mechanisms [17, 32]. In particular, Lys187 is well known to interact with the 3-hydroxyl group of 6PG [36].

Docking simulations of the substrate were also performed to predict the substrate-binding mode. The 6PG molecule fitted the substrate-binding cavity well, which was predicted based on other similar structure (Fig. 3B). In the substrate-binding pocket, the N-term domain, CD, C-term domain were involved in the binding of substrate. The phosphate of 6PG interacted with Tyr195, Lys260 and Arg287 in the CD; and with Arg446 in the C-term domain of another chain (Figs. 3B and 3C).

The catalytic residues of 6PGD are well known, *i.e.*, Ser, His, Asn, and their corresponding positions in other 6PGD are conserved. As a result of the structural comparison with *Oa*6PGD (PDB code : 1PGP), we determined that Ser133, His190 and Asn191 were catalytic residues of *Cg*6PGD (Fig. 3D) [37].

### NADP-Binding Site

**Fig. 4:**
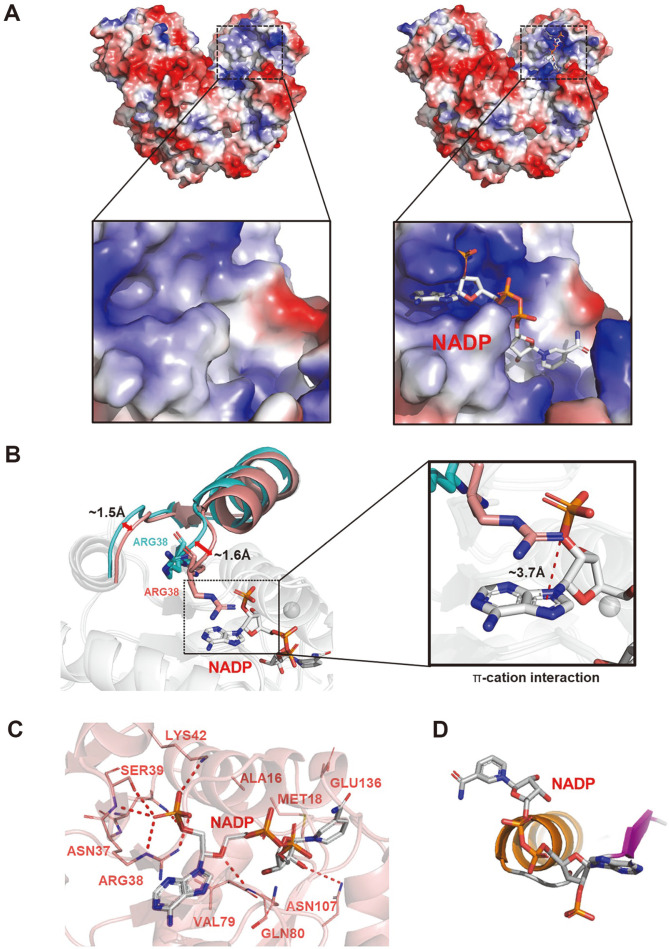
Co-factor (NADP)-binding model of *Cg*6PGD. (**A**) Electrostatic potential surface of the *Cg*6PGD_apo (left) and *Cg*6PGD_NADP (right) forms. NADP molecules are shown as gray-colored stick models. (**B**) Conformational changes in *Cg*6PGD_NADP. NADP is presented as a sphere model. The difference in loop distance is labeled. (**C**) Co-factor (NADP)- binding model of *Cg*6PGD. The structure of *Cg*6PGD is shown as a cartoon diagram, and the residues that bind to NADP are presented as stick models and labeled. (**D**) Pyrophosphate of the NADP-binding form.

To characterize the conformational change between *Cg*6PGD_apo and *Cg*6PGD_NADP, we determined the crystal structures of both forms (Fig. 4A). Structural differences between *Cg*6PGD_apo and *Cg*6PGD_NADP were detected in the residual movement together with the loop (Fig. 4B). These residues moved 1.5 and 1.6 Å closer to NADP from their positions, respectively.

A NADP-binding site was observed in the N-term domain. The NADP-binding site was constructed by Ala16, Met18, Asn37, Arg38, Ser39, Lys42, Val79, Gln80, Asn107 and Glu136. Among these residues, Arg38, Ser39 and Lys42 were crucial for binding to NADP (Figs. 1B and 4C). Arg38 is well known as a crucial residue for specificity of the enzyme for NADP [17]. The guanidinium group of Arg38 established a stable π -cation interaction with the adenine ring of NADP, and the π-cation interaction and hydrogen bonds that formed between Arg38 and NADP played an important role in the stable cofactor binding of *Cg*6PGD (Fig. 4B) [38, 39]. The nicotinamide ring of NADP which is involved in the redox reaction, and 6PG are located close together [40]. Asn107 interacts with 6PG and cooperates with Met18 to form a shallow cavity for NADP-binding. Moreover, these residues helped to form closed structure by holding the adenosine ribose. Via this reaction, the nicotinamide ring of NADP interacts with the catalytic triad, *i.e.*, Ser133, His190 and Asn191 [17].

A structural comparison between the docking simulation of 6PG and *Cg*6PGD_NADP showed that the nicotinamide ring of NADP and the carboxylate group of 6PG are positioned in parallel, to achieve electron transfer [41]. By checking our *Cg*6PGD_NADP-binding form, we found that *Cg*6PGD had class I that is typical of NAD(P)-binding proteins (Fig. 4D). Val12-Phe26 of *Cg*6PGD is the consensus sequence of the class I 3d motif,[VILF]-X-G-X-[GSA]-X_2_-[GAS]-*X*_6_-[LAIFWCG], which is the most frequent in NAD(P)-binding proteins [42].

### Enzymatic Kinetics and Relative Activity

**Fig. 5:**
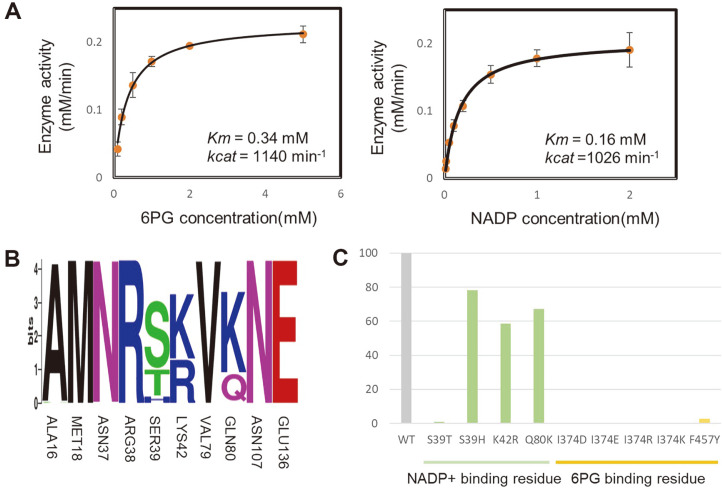
Enzymatic kinetics of *Cg*6PGD. (**A**) Kinetic analysis of *Cg*6PGD. The reaction velocity was plotted vs. the substrate (6PG) concentration (left) and co-factor (NADP) concentration (right) based on the Michaelis-Menten equation. The experiments were performed in duplicate and the standard deviation is indicated by the error bar. Various concentrations of 6PG (0.1~5 mM) and NADP (0.01~2 mM) were used. (**B**) Conservation analysis of reisudes associated with NADP-binding. (**C**) Relative activity of *Cg*6PGD. The activity value of the mutants is expressed with the wild-type form set at 100%. The green and yellow graphs present the relative activities of the NADP-binding residue and 6PG-binding residue, respectively.

The enzymatic kinetic parameters for checking substrate and NADP affinity were obtained by measuring the initial velocity of *Cg*6PGD under various substrate and NADP concentrations, respectively. The enzymatic kinetics graph of *Cg*6PGD obeyed the Michaelis-Menten kinetics. The *K_m_* and *k_cat_* values are 0.34 mM and 1,140 min^–1^ in the presence of varying substrate concentrations. The *K_m_* and *k_cat_* values of the NADP were 0.16 mM amd 1,026 min^–1^, respectively (Fig. 5A and Table 2).

A conservation analysis was performed using WebLogo to confirm the conservation of the NADP binding residue. Among the residues that were involved in binding of NADP, Ser39, Lys42, and Gln80 were not conserved in 705 homologous sequences (Fig. 5B). To check the effects of residues regarding NADPH activity, the relative activity is evaluated (Fig. 5C). In the case of Ser39, S39T showed little activity, and S39H showed a 22% activity decrease were detected compared with the wild-type (WT) form. The analysis of the structure of *Cg*6PGD_NADP made it appearent that Thr and His prevented the binding of NADP. Regarding Lys42, it showed an almost equal ratio with Arg in the 705 homologs. However, K42R also exhibited a halved activity vs. the WT. Arg is longer than Lys; thus, it may interrupt the binding to NADP. Interestingly, in the case of Gln80, Lys was more than Gln in 705 homologs. However, the activity showed a 32% decrease in Q80K. Because Lys is longer than Gln, it seems to interfere with the binding of NADP. As a result of measuring residues which are binding NADP by changing the unconserved residues in 705 homologous (S39T, S39H, K42R, Q80K), the activity of the residues of WT was higher. These results imply that *Cg*6PGD might carry the most effective residues in its active site. An assessment of the properties of the residues located in 6PG-binding site was also performed. Ile374 and Phe457 were the non polar residues that bound with 6PG, and these residues were situated near the 6PG-binding site. Considering that the activity of I374D, I374E, I374R, I374K and F457Y were closer to zero compared to the WT, non polar residues are expected to be important for binding 6PG.

In summary, we determined the crystal structures of the apo and complex forms of *Cg*6PGD with a resolution of 2.4 and 1.9 Å, respectively; *Cg*6PGD is the crucial enzyme in NADPH production. By comparing it with other 6PGD structures and amino acid sequences, we showed that most 6PGD enzymes had a very similar overall structure and conserved sequences. The substrate and NADP-binding sites were located in close proximity, and Asn107 and Met18 cooperated to form a shallow cavity. Site-directed mutagenesis revealed that the residues of *Cg*6PGD were preferred for the efficient production of NADPH. Our structural analysis of *Cg*6PGD may contribute to the improvement of NADPH production and enhance many microbial-based industries.

## Figures and Tables

**Table 1:** Data collection and refinement statistics of *Cg*6PGD apo and complex form.

	*Cg*6PGD_apo	*Cg*6PGD_NADP
Data collection		
Space group	P 2_1_ 2_1_ 2_1_	P 2_1_ 2_1_ 2_1_
Cell dimensions		
*a, b, c* (Å)	63.90 120.30 152.60	64.00 119.47 153.45
α, β, γ ()	90.00 90.00 90.00	90.00, 90.00, 90.00
Resolution (Å)	50.00-2.44	50.00-1.93
*R*_sym_ or *R*_merge_	12.1 (36.9)	6.5 (33.8)
*CC_1/2_*	0.978 (0.773)	0.992 (0.93)
*I / σ (I)*	15.9 (3.18)	35.667 (6.865)
Completeness (%)	94.9 (92.4)	99.0 (97.4)
Redundancy	3.2 (2.5)	5.5 (5.5)
Refinement		
Resolution (Å)	33.99-2.41	33.84-1.90
No. reflections	41803	87822
*R*_work_ / *R*_free_	19.951 / 26.708	16.408 / 20.217
No. atoms	7314	8011
Protein	7238	7218
Ligand/ion	0	143
Water	76	650
*B*-factors	39.794	22.082
Protein	42.582	22.903
Ligand/ion	0	44.127
Water	35.828	30.075
R.m.s. deviations		
Bond lengths (Å)	0.008	0.011
Bond angles (°)	1.615	1.643
PDB ID	8I4N	8I4Q

**Table 2:** Kinetic analysis of *Cg*6PGD for 6PG/NADP.

Kinetic parameters	6PG			NADP		
6PG/NADP cofactor	*k*_cat_ [min^-1^]	K_M_ [mM]	*k*_cat_/K_M_ [(mM min)^-1^]	*k*_cat_ [min^-1^]	K_M_ [mM]	*k*_cat_/K_M_ [(mM min)^-1^]
Wild type	1139.55 ± 27.1	0.34084 ± 0.03	3343.358	1027.35 ± 16.9	0.16276 ± 0.0095	6312.055
